# A novel hypothesis for atherosclerosis as a cholesterol sulfate deficiency syndrome

**DOI:** 10.1186/s12976-015-0006-1

**Published:** 2015-05-27

**Authors:** Stephanie Seneff, Robert M. Davidson, Ann Lauritzen, Anthony Samsel, Glyn Wainwright

**Affiliations:** Computer Science and Artificial Intelligence Laboratory, MIT, Cambridge, MA 02139 USA; Internal Medicine Group Practice, PhyNet, Inc, 4002 Technology Center, Longview, TX 75605 USA; Independent Researcher, Houston, TX 77084 USA; Research Scientist and Consultant, Deerfield, NH 03037 USA; Independent Reader of Research, Leeds, UK

## Abstract

**Background:**

Despite a vast literature, atherosclerosis and the associated ischemia/reperfusion injuries remain today in many ways a mystery. Why do atheromatous plaques make and store a supply of cholesterol and sulfate within the major arteries supplying the heart? Why are treatment programs aimed to suppress certain myocardial infarction risk factors, such as elevated serum homocysteine and inflammation, generally counterproductive?

**Methods:**

Our methods are based on an extensive search of the literature in atherosclerotic cardiovascular disease as well as in the area of the unique properties of water, the role of biosulfates in the vascular wall, and the role of electromagnetic fields in vascular flow. Our investigation reveals a novel pathology linked to atherosclerosis that better explains the observed facts than the currently held popular view.

**Results:**

We propose a novel theory that atherosclerosis can best be explained as being due to cholesterol sulfate deficiency. Furthermore, atheromatous plaques replenish the supply of cholesterol and sulfate to the microvasculature, by exploiting the inflammatory agent superoxide to derive sulfate from homocysteine and other sulfur sources. We argue that the sulfate anions attached to the glycosaminoglycans in the glycocalyx are essential in maintaining the structured water that is crucial for vascular endothelial health and erythrocyte mobility through capillaries. Sulfate depletion leads to cholesterol accumulation in atheromas, because its transport through water-based media depends on sulfurylation. We show that streaming potential induces nitric oxide (NO) release, and NO derivatives break down the extracellular matrix, redistributing sulfate to the microvasculature. We argue that low (less negative) zeta potential due to insufficient sulfate anions leads to hypertension and thrombosis, because these responses can increase streaming potential and induce nitric-oxide mediated vascular relaxation, promoting oxygen delivery. Our hypothesis is a parsimonious explanation of multiple features of atherosclerotic cardiovascular disease.

**Conclusions:**

If our interpretation is correct, then it would have a significant impact on how atherosclerosis is treated. We recommend a high intake of sulfur-containing foods as well as an avoidance of exposure to toxicants that may impair sulfate synthesis.

## Background

After many years of research, atherosclerosis and the associated risk of myocardial infarction (MI), the number one cause of death worldwide, remains a well-studied but poorly understood condition. Several risk factors have been identified, such as elevated serum low density lipoprotein (LDL), low cholesterol content in high density lipoprotein (HDL), elevated serum homocysteine, diabetes, left ventricular hypertrophy, etc. [1], but it remains controversial whether these risk factors are merely associative or actual causes. Particularly troublesome is the paradoxical reverse epidemiology profile of people suffering from end stage kidney disease [2] and other chronic inflammatory conditions such as systemic lupus erythematosus [3]. Here, the normal risk factors – obesity, high blood pressure, high cholesterol, and high homocysteine – all become protective factors in heart disease risk. And these patients have extremely high risk of heart disease (this is in fact their most common cause of death) [4], despite the inversion of risk factors.

Increasingly, atherosclerosis is framed as an inflammatory condition [5–7], and clearly inflammation plays a critical role in vascular dysfunction. Ischaemic tissue will face necrosis if it is not reperfused, yet reperfusion can also lead to severe tissue damage due to oxidative attack [8]. Inflammation is the one factor that is strongly linked to cardiac disease in association with kidney failure [9, 10]. Others have proposed that atherosclerosis is an infectious disease, as infective agents, especially *Chlamydia pneumoniae* and Herpes virus, have been found consistently in association with atherosclerotic plaque regions [11, 12].

## Results and discussion

In this paper we present an alternative view of atherosclerosis as a set of responses aimed at correcting disturbances in biological water structure caused by depletion of crucial biosulfates – especially sulfurylated^1^^1^ cholesterol and glycosaminoglycans (GAGs) -- in the cardiovascular system. Evidence for a role of sulfur deficiency in atherosclerosis will be surveyed in (Evidence for a role of sulfur deficiency in atherosclerosis below). In Sections (The Exogenous Interfacial Water Stress (EIWS) theory of inflammation, Electrokinetic vascular streaming potential and Biosulfates as enablers of healthy blood flow), we consider the biophysics of structured water and vascular streaming potential and the importance of sulfates in maintaining them and in enabling healthy blood flow. In Section (Extracellular matrix sGAG turnover as a mechanism for sulfate redistribution), we discuss how sulfurylated GAG turnover in the vascular system may be used to redistribute sulfate to replenish cholesterol sulfate levels in the capillaries. We will then turn our attention in Section (Glutathione and GGT) to emergency routes -- with damaging side effects – by which the body may replenish vascular sulfate supplies by conversion of less-oxidized sulfur compounds such as glutathione, H_2_S, and homocysteine to sulfate.

### Evidence for a role of sulfur deficiency in atherosclerosis

While many research papers have described in detail the processes that take place in the coronary artery wall in association with the accumulation of fat and cholesterol, few have attempted to explain *why* these processes are happening. It is particularly surprising that the arteries supplying the heart, arguably the most important organ in the body, should be especially vulnerable to fatty deposits.

In this section we will review the evidence for a role of sulfur deficiency in atherosclerosis. Table 1 provides a chronological list of key research and review papers pointing strongly to involvement of a variety of oxidized and reduced sulfur compounds in this condition. These articles will be discussed in more detail at various points throughout this paper.

**Table 1:** Timeline of significant research papers linking atherosclerosis with impaired sulfate supply to the vasculature

Publication	Key finding
Mann, 1960 [13]	Sulfur supplementation protects monkeys from atherosclerosis, due to dietary cholesterol and choline enrichment.
Hauss et al., 1962 [110]	Chondroitin sulfate incorporation into connective tissue is greatly enriched in the atherosclerotic aorta.
McCully, 1969 [14]	Homocysteine plays an important role in arteriosclerosis.
Bleau et al., 1974 [84]	Deficient Ch-S in RBCs leads to increased hemolysis; Ch-S stabilization of RBC membrane by SEM.
Bergner et al., 1981 [32]	Cholesterol sulfatase deficiency leads to massive accumulation of Ch-S in RBCs and plasma.
Avila et al., 1996 [37]	Chagas infection induces Ch-S autoantibodies.
Paka et al., 1999 [98]	ApoE promotes both cholesterol egress from macrophages in the plaque and sulfation of the glycocalyx.
Strott, 2003 [30]	The only review article available on Ch-S’s role in physiology.
Freitas et al., 2005 [38]	Chagas disease induces heart failure many years later; with minimal evidence of cardiovascular disease.
Ren et al., 2007 [35]	Ch-S regulates lipid metabolism in the liver.
Ma et al., 2008 [34]	Ch-S decreases lipid biosynthesis in macrophages.
Qiao et al., 2010 [141]	H_2_S is involved in pathogenesis of atherosclerosis.
Davidson and Seneff, 2012 [49]	Sulfate deficiency leads to decreased deformability and increased aggregation of RBCs, increased (less negative) zeta potential, and increased capillary surface tension.
Manna and Jain, 2011 [118]	H_2_S enhances glucose uptake by cells
Seneff et al., 2012 [16]	eNOS produces sulfate catalyzed by sunlight.
Xu et al., 2012 [33]	Ch-S suppresses inflammatory response in macrophages.
Xu et al., 2014 [142]	H_2_S is a promising therapy for atherosclerosis.
Toshikuni et al., 2015 [130]	Elevated serum GGT predicts carotid plaque build-up.

Interesting early studies on the New World primate, *Cebus fatuella*, revealed that a condition strongly resembling atherosclerosis could be produced in the artery wall by feeding these monkeys a high fat, high cholesterol, high choline diet [13]. However, this effect could be eliminated or drastically reduced if the monkeys were simultaneously supplemented with sulfur-containing amino acids. Children with disorders of cysteine metabolism leading to excess serum homocysteine develop arterial damage at an early age that is reminiscent of atherosclerotic cardiovascular disease [14]. The consumption of garlic is inversely correlated with the progression of atherosclerosis, and, in [15] it was argued that the protective benefit is due to the ability of human erythrocytes to convert garlic-derived polysulfides into hydrogen sulfide gas. These observations and others strongly support the model that impaired sulfur supplies to the vasculature plays a critical role in atherosclerosis.

In [16], the argument was developed that atherosclerosis results from a deficiency in a critical nutrient, cholesterol sulfate (Ch-S), and that the atheroma is a locale where endothelial cells, macrophages, and platelets collaborate to produce Ch-S from homocysteine and oxidized LDL. It was argued that an underlying deficiency in Ch-S, due principally to insufficient dietary sulfur and inadequate sun exposure to the skin, leads, over time, to severe depletion of cholesterol and sulfate in the tissues. This deficiency becomes life-threatening with age, and atherosclerotic plaque is a well-choreographed program for renewal of Ch-S. Unfortunately, there is necessarily collateral damage from superoxide exposure, as superoxide is required to oxidize the LDL and to catalyze the reaction that produces sulfate from homocysteine thiolactone [17].

Worldwide geographical data show an inverse relationship between cardiovascular disease and annual sunlight availability [18]. In a study conducted in the British Isles, 49 % of the variance in mortality from coronary heart disease was accounted for by mean annual sunshine hours as measured by the Meteorological Office [19]. However, placebo-controlled trials failed to show any benefit from vitamin D3 supplementation [20]. We propose that the benefit comes from Ch-S synthesis instead. In [16], it was proposed that the protein endothelial nitric oxide synthase (eNOS), along with sunlight, catalyzes sulfate production in erythrocytes, endothelial cells, platelets and keratinocytes in the skin. Thus, eNOS is a dual-purpose enzyme, producing sulfate when it is membrane–bound and producing nitric oxide when it is free in the cytoplasm. We hypothesize that the overuse of sunscreen has played a dual damaging role not only because sunlight catalysis is suppressed but also because the aluminum found in high-SPF sunscreens as an emulsifier actively disrupts eNOS’ function [21]. eNOS is an orphan cytochrome P450 (CYP) enzyme [22], and aluminum is a known disruptor of CYP enzyme function through its displacement of the iron in the heme group [23]. Many other environmental toxicants also disrupt CYP enzymes, including mercury [24, 25], arsenic [26], cadmium [24], glyphosate [27, 28], and lead [25, 29].

There exist a large number of different sulfatases and sulfotransferases that effect sulfuryl group transfer from a source molecule to a target molecule, with an ATP-activated form of sulfate, called 3′-phosphoadenosine-5′-phosphosulfate (PAPS) serving as an intermediary [30, 31]. Sulfotransferase deficiency is associated with remarkable accumulations of Ch-S in red blood cells (7500 μgrams/100 ml vs < 300 in controls) and in plasma (3300 vs. < 350) [32]. This implies that Ch-S produced in erythrocytes and the endothelial wall is essential for distributing both cholesterol and sulfate to the tissues.

The nuclear receptor peroxisome proliferator-activated receptors (PPARs) regulate both lipid metabolism and the inflammatory response in macrophages in the atheroma. A recent experiment showed that 25-hydroxycholesterol sulfate (25HCS) activated PPARγ in macrophages, resulting in the suppression of NF-κB and the subsequent inflammatory response [33]. 25HCS also decreases lipid biosynthesis in both macrophages [34] and the liver [33, 35]. The unsulfurylated form of the molecule had the opposite effect. This supports the idea that inflammation is needed for sulfate synthesis and that sulfate is needed for cholesterol export.

MI is an acute response to interruption of the blood supply to a part of the heart, usually assumed to be caused by the rupture of vulnerable plaque occluding the artery wall. However, in about 10 % of patients, there is no apparent plaque accumulation associated with the acute event. Despite this apparently healthy arterial profile, such individuals experience a similar incidence of death and recurrent MI in long-term follow-up, compared with patients with severe residual stenosis [36]. A most interesting case in this regard is the myocardial dysfunction associated with Chagas disease [37]. *Trypanosoma cruzi* (Chagas disease) is a parasitic infection that occurs predominantly in South America, where an individual infected early in life experiences a substantially increased risk to premature heart failure many decades later [38, 39]. While there is a substantial impairment in heart function associated with Chagas disease, including cardiomyopathy and left ventricular hypertrophy, people with this condition are remarkably free from atherosclerotic plaque build-up.

We propose that this unusual profile is due to the fact that *T. cruzi* produces a steroid that remarkably resembles Ch-S. A study revealed that 86 % of the Chagas patients tested had developed autoantibodies against Ch-S – a very specific effect in that closely related steroid sulfates were not affected [37]. Thus, the atheroma is unable to function in the normal role of producing Ch-S, due to the autoimmune reaction this would provoke. As a consequence, the heart develops severe cumulative impairment over time, due to deficiencies in cholesterol and sulfate supplies, ironically caused by the absence of atherosclerosis.

The most precarious phase of an MI is reperfusion rather than ischemia, as reperfusion injury can be extensive and life-threatening [40]. Reperfusion injury is associated with systemic exposure to inflammation from superoxide and its reaction products, which is believed to be the principle source of injury [40, 41]. Ischemic reperfusion is characterized by a complex sequence of events leading in extreme cases to systemic inflammation and edema, microvascular dysfunction (the “no flow” phenomenon) and multiple organ failure, the leading cause of death in the critically ill [41].

Thus, a local blockage, for example due to thrombosis or traumatic injury, whether in the heart or elsewhere, can lead, through complex signaling cascades, to a systemic response – a catastrophic failure in the blood transport system – which appears to be the ultimate cause of death in these cases. Most interesting, however, is the observation that suppression of the systemic response through drug intervention results in a substantial *worsening* of tissue damage via extensive apoptosis at the local site [42]. Thus, the systemic response can lead to a positive outcome: it produces some product that somehow protects cells at the site of injury from apoptosis.

In addition to Ch-S, the sulfurylated glycosaminoglycans (GAGs) that comprise the so-called glycocalyx structures lining the endothelial lumen also appear to contribute importantly to cardiovascular health [43]. These sGAGs contain linear chains of modified sugar molecules such as heparan sulfate and chondroitin sulfate, which can be either attached to membrane-bound proteins or free floating. Pericytes are a specialized cell type that has gained attention in recent years due to the realization that they are essential for the health of capillaries, arterioles and venules, and that they are particularly prevalent in the brain [44]. Pericytes are situated in the microenvironmental niche of the arteriole or venule wall, and they differentiate into different cell types in response to environmental stress signals, to assist with tissue regeneration and repair [45]. Pericytes wrap around endothelial cells and are in close communication with them through junctional complexes. The migration of pericytes depends on paracrine signaling from growth factors like vascular endothelial growth factor (VEGF) and platelet derived growth factor (PDGF). In [46], it was shown that the PDGF-mediated recruitment of pericytes to the capillary wall critically depends on sufficient N-sulfurylation of heparan sulfate proteoglycans (HSPGs) in the glycocalyx. N-deacetylase/N-sulfotransferases (NDSTs) are essential for the sulfurylation process [47], and mice with a deficiency in NDST-1, the major N-sulfurylating enzyme [48], often die in utero, or live for just a few days following birth. Microscopic examination of the brain vasculature revealed severe depletion in pericytes.

The contribution of sGAGs to cardiovascular health rests on their ability to maintain specially-structured water zones in the blood vessels. This topic will be explored in more detail in the following three sections.

### The Exogenous Interfacial Water Stress (EIWS) theory of inflammation

As noted above, atherosclerosis is increasingly regarded as an inflammatory condition [5–7]. In earlier reviews, Davidson and Seneff have proposed exogenous interfacial water stress (EIWS) – the disruption of normal interfacial tension between water and biomacromolecules *in vivo* by exogenous agents such as the aluminum aquo cation -- as the earliest step in the pathogenesis of inflammation and disease [49, 50].

In this section, we will focus on biophysical properties of interfacial water that are relevant for blood flow in the vascular system. In the next section, we will discuss streaming potential, and its important role in inducing nitric oxide synthesis. We will follow this with a section that considers in more detail the roles played by biological sulfate species, including sGAGs and Ch-S, in maintaining healthy interfacial water structure and enabling blood flow.

A full description of the complex biophysics of dynamically-structured water and its electrokinetic properties is beyond the scope of this paper. However, several recent review articles can be cited [51–54]. It has become abundantly clear that water is an active and even key participant in chemical reactions, and is, furthermore, the natural environment for biochemical processes [54]. Moreover, the interaction of water with the solutes and surfaces encountered in biological systems at relatively close range results in water properties that vary substantially from those measured for the pure bulk liquid.

In 2012, Davidson and Seneff [49] presented for the first time a number of novel biophysically-based hypotheses as to the role of interfacial water, sunlight, and the biosulfates in facilitating and enabling microvascular blood flow and gas exchange. The concept of surfactant-induced interfacial water stress was presented, and the conclusion was reached that the initial events in the inflammatory cascade are purely biophysical. The role of the bio-sulfates in maintaining cell membrane function and the role of endothelial nitric oxide synthase-derived nitric oxide in acute shock and Sudden Death Syndrome were described. The concepts, hypotheses, and conclusions in this paper provide building blocks upon which future hypotheses can be generated and tested.

In a series of *in vitro* experiments, Pollack and coworkers have established that water adjacent to hydrophilic surfaces (interfacial water) differs dynamically from bulk water in several respects, including exhibiting up to ten times higher viscosity [55, 56]. Guo and Friedman [57] and Tielrooij et al. [58] have provided data to support an earlier prediction by Ling [59, 60] that even small organic molecules and solutes can induce formation of “polarized oriented multilayers” of interfacial water.

Pollack and coworkers proposed the term “exclusion zone” for this interfacial water, based on the observation that solutes and suspended particles were excluded from this water at distances up to a few hundred microns from the hydrophilic surface [56]. He proposes that the structured water along biological membranes forms hexamers [61] that closely resemble the structure of ice. He wrote: “We found the honeycomb sheet model more promising, its hexamers lying out of register with those of adjacent sheets. This model could account for the EZ's net charge; plus, it has the advantage of precedent because of its similarity to ice.” ([56], p. 68). Most likely, the exclusion zone is built of such highly structured “liquid crystals” and it induces a charge separation with a voltage drop across the interface with the surrounding bulk water, thus providing a battery-like source of energy.

Del Giudice has compared this interfacial or exclusion zone (EZ) water with Preparata’s proposed water “coherence domains” (CDs), derived via a quantum electrodynamic application of quantum field theory [62]. In earlier work, Preparata also likened water to a plasma in the sense that it contains a roughly equal number of positively and negatively charged particles and thus can conduct electricity and be influenced by magnetic fields [63]. More recently, in a proposed continuum solvent model, Duignan et al. have also described interfacial water as a coherent, plasma-like phase [64].

In addition to the evidence reported for special, “non-bulk” water zones near hydrophilic surfaces [56], neutron reflectivity measurements of Doshi et al. have revealed reversible changes in the size of a reduced-density water region near a model hydrophobic surface, depending on the amounts and kinds of dissolved gases present in the water [65]. Their observation of a decrease in size of the lower-density region in the water as gas concentration was reduced explains the hydrophobic effect and its reportedly lower magnitude in degassed water [65]. Shatalov, Bunkin, and others have published data pointing to an important role for ion-stabilized nanobubbles in modulating interfacial water tension [53, 66, 67]. These studies of the effects of dissolved gases on interfacial water properties provide important insights into the important role of dissolved gases, such as NO, CO, and H_2_S, as vascular signaling molecules [68]. This subject will be discussed further in Section (Electrokinetic vascular streaming potential) below.

From this brief survey, we can see that essentially all types of surfaces and solutes found in biological systems have important interactions with water that influence interfacial water structure and tension. The sulfur-containing surfaces and solutes of particular interest for maintaining healthy water properties in blood vessels include the sGAGs that, as mentioned above, line the endothelial lumen, and Ch-S, which is present in all cell membranes and, at low levels, in blood plasma [30]. David Grant’s group has provided infrared and NMR spectroscopic evidence to support the view that heparan sulfate proteoglycans (a type of sGAG) impart structure to water *in vivo* [69]. Importantly, results of infrared photodissociation spectroscopy studies have provided evidence of relatively long-range water ordering properties for sulfate anions [70].

**Fig. 1:**
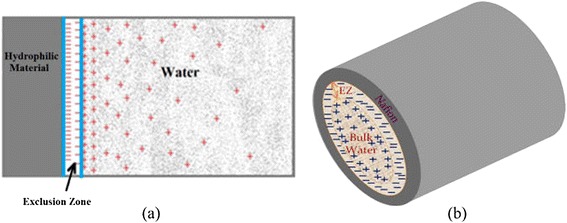
**a**. Distribution of charge in the exclusion zone and water beyond the exclusion zone as shown by electrical potential measurements and pH- sensitive dye studies. Protons spread in bulk water, although some cling to the negatively charged EZ. **b** Disposition of charge anticipated in a tubular configuration. (Reproduced from Rohani and Pollack, 2013 [72], with permission from the American Chemical Society. Copyright © 2013)

**Fig. 2:**
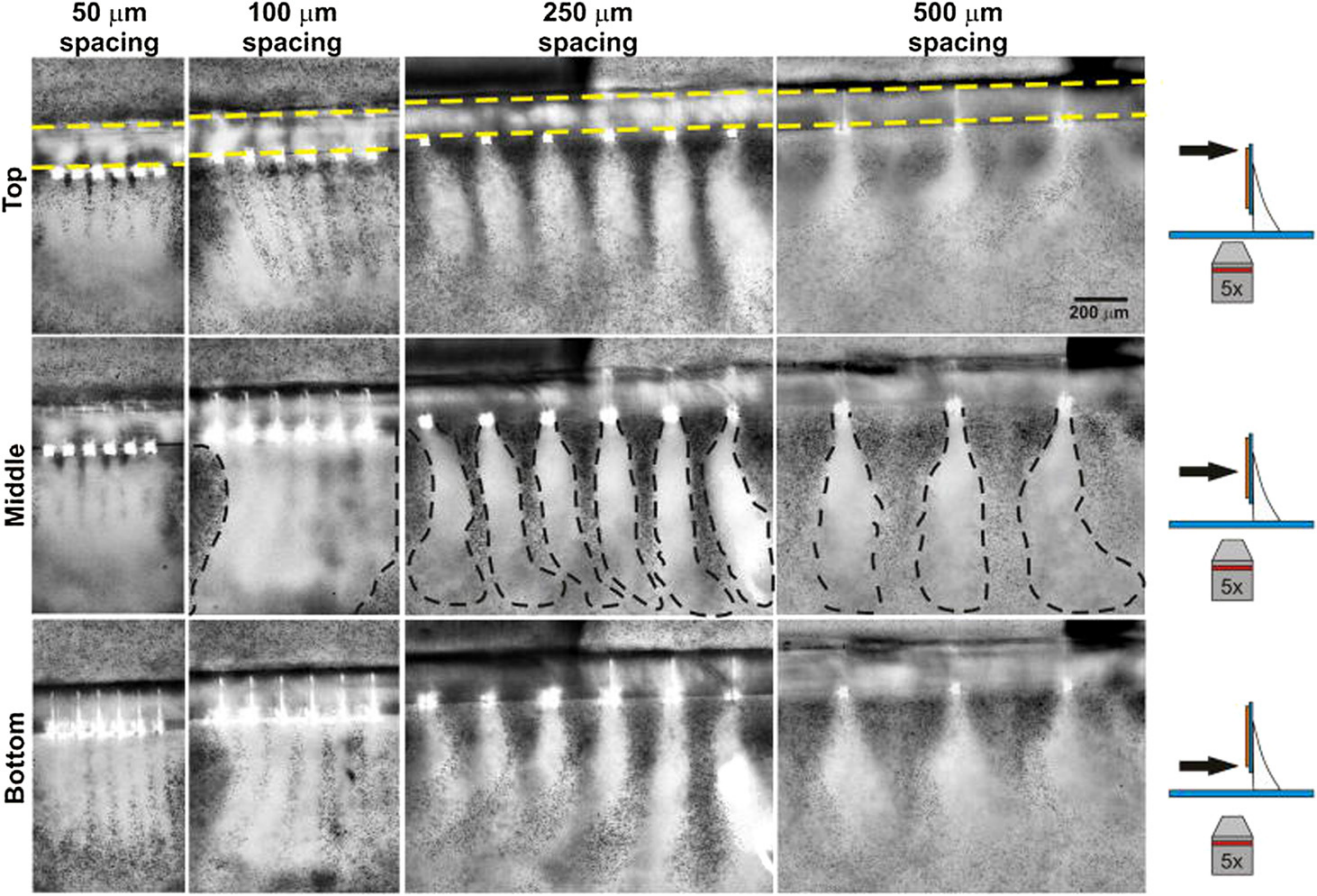
Nafion^R^ strips spaced at 50, 100, 250 & 500 μm edge-to-edge. Exclusion zones form from the Nafion^R^ surfaces and merge into an unbroken EZ in 50- and 100 μm-spaced lines. Nafion^R^ strips are seen as the bright white squares on the images. At 250 and 500 μm the EZ forms in discrete zones that do not combine. The yellow dashed lines demarcate edges of glass support. Dashed black lines indicate extent of clear EZ. Diagram on the far right indicates the viewing height (arrow) on which the objective was focused. (Reproduced from Figueroa and Pollack (2011) [73] with permission from WIT Press)

Moreover, one of the hydrophilic surfaces used prominently in Pollack’s studies of EZs comprised sheets of the sulfonate-rich fluoropolymer Nafion^R^ [55, 56, 71]. Recent studies of Pollack and coworkers with potential high relevance to water behavior in blood vessels include observations on water flow through submerged Nafion^R^ tubes [72] and elegant work on EZ formation near discontinuous Nafion^R^ surfaces [73]. Figure 1 depicts the capillary glycocalyx and shows how the Nafion^R^-based tubes can model its behavior. Figure 2 provides physical evidence of the structured water adjacent to Nafion strips, which draws parallels with the architecture of the glycocalyx.

The observations of Bunkin *et al.* of changes in electrostatic potential and the refractive index of water provide additional evidence of a separate phase of water near Nafion^R^ surfaces [74]. In addition, Zhao *et al.* have noted unexpected effects of light on self-assembly of colloidal, sulfate-decorated particles near larger beads in water [75].

### Electrokinetic vascular streaming potential

In this section we consider the effects of interfacial water on blood flow in the vascular system. The pumping action of the heart generates pressure that pushes blood through blood vessels. This pressure-induced flow of the blood past the charged sGAGs lining the endothelium generates an electric streaming potential. We will focus on considering a recent paper by Trivedi et al. [76], which demonstrated that the electrokinetic vascular streaming potential (EVSP) caused by blood flow has significant biological effects on endothelial cells [76]. This paper may have solved in one stroke of genius the mystery of why both elevated blood pressure and elevated heart rate might have beneficial effects [76]. In fact, EVSP may be the most important contributor to vascular health.

A key insight is that the blood is filled with negatively charged “particles” in colloidal suspension, which includes both cells such as red blood cells (RBCs) and platelets, as well as lipid particles such as LDL and HDL, serum proteins such as albumin and γ globulins, and fragments of sulfurylated polysaccharides like heparan sulfate. A moving charged particle creates an electromagnetic field, which can be used as a signaling device in biological systems. What Trivedi et al. [76] have shown is that endothelial cells respond to these electromagnetic signals by producing nitric oxide, which has been identified as the endothelial-derived relaxing factor (EDRF) in arteries and veins [77].

The signal has both a DC and an AC component. The AC component is synchronous with the heart rate, as the suspended particles accelerate and decelerate in response to the force field of the beating heart. The paper showed that there was a significant (nonlinear) increased response to a 2Hz signal (corresponding to a heart rate of 120 beats per minute) compared to a 1Hz signal (60 bpm). Hence, increasing heart rate has a direct effect of inducing increased NO production, and therefore vascular relaxation [77].

**Fig. 3:**
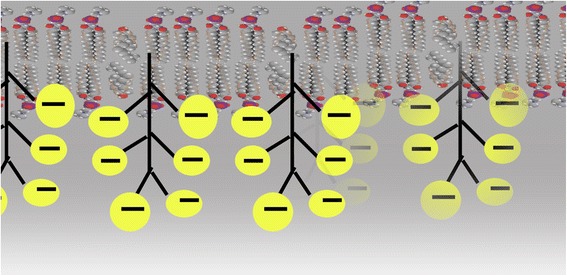
Schematic of the bottle brush structure of the glycocalyx, including the lipid bilayer at the top, and the branching trees of glycosylated proteins with sulfates in the heparan sulfate chains providing negative charge and kosmotropic effects on the surrounding water, with strong analogies to Pollack’s Nafion^R^ strips experiments shown in Fig. 2

Figure 3 provides a schematic of the principle that vascular flow is critically dependent on the negative charge provided by sulfate ions attached to the glycosaminoglycans in the capillary wall and to cholesterol in the plasma membrane of red blood cells. Electrostatic repulsion prevents RBCs from adhering to the capillary wall, and the encasement of the glycocalyx within structured water, formed in response to the kosmotropic properties of sulfate, provides a smooth surface further protecting from adhesion. The RBCs are propelled toward the venous end of the capillary by the electromagnetic field induced by the voltage drop across the capillary. The RBCs themselves act as moving charged particles that create a circular magnetic force around the capillary wall. This force induces nitric oxide release from the endothelial cells, which promotes vascular relaxation and increases flow.

The experimental results presented in [76] virtually prove, by process of elimination, that structured water in the interphase of the endogenous biofilm of the endothelial glycocalyx is the “target” of the EVSP. Based in substantial part on Trivedi et al. [76], we propose the hypothesis that interfacial water is also the target of extremely low frequency electromagnetic (ELF EM) energy from the environment, *in vivo*, including that energy afforded by the EVSP to the interfacial water found in the EGL of the vascular endothelium, systemically, including that of the heart.

We find to be quite striking the interpretations derived from the seminal experiments in the Trivedi [76] paper, which demonstrated that the effect of EVSP on the NO signal is not simply tied to the depolarization effect on the endothelial cells. They propose several sites of action of the ELF fields on the pathways that are directly responsible for producing the NO signal in the presence of extracellular calcium. Their experiments with ELF electrical stimulation under calcium-free solutions show increases in NO production in an amplitude- and frequency-dependent manner. The conclusion is that the field itself acts on a target that potentiates NO production without requiring calcium flux.

Having excluded many of “the usual suspects” as targets of the ELF EM field of the EVSP, including those associated with calcium flux, Trivedi et al. suggest that “the likely mechanism that links these fields to cellular effects is the electroconformational coupling of an applied periodic electric field to membrane-associated processes, which has been established both theoretically and experimentally.” [76] Of course, this view is fully compatible with the EIWS theory, in which an underlying tenet is that protein structure and function are “slaved” to biological water dynamics [78], and effectively invites our proposal that a logical candidate for the biological target of the ELF EM energy of the EVSP is the interfacial water itself.

The classical formula for streaming potential, known as the Helmholtz-Smoluchowski (HS) equation, is very enlightening, because its magnitude is proportional to zeta potential and blood pressure, and inversely proportional to blood viscosity. Zeta potential, in turn, depends on the amount of negative charge around the charged particles. According to A.V. Delgado et al. [79], electrokinetic or zeta-potential, ζ, is identified as the potential at the plane where slip with respect to bulk solution is postulated to occur ([79], p. 1759). When RBCs are deficient in Ch-S in their membranes, this will be reflected in a lowered (less negative) zeta potential. This can be counterbalanced by increasing the blood pressure to compensate, which could be the main beneficial effect of high blood pressure, a so-called “pathological” condition associated with cardiovascular disease.

Furthermore, viscosity, the denominator term in the HS equation, can also be manipulated by reducing the total count of RBCs in circulation. This will have a significant effect in reducing viscosity, thus increasing EVSP. This may be the source of the essential anemia associated with certain end-stage diseases like kidney failure and cancer [80, 81]. eNOS dysfunction is a known factor in kidney disease [82], and we propose that this has to do with insufficient sulfate synthesis by eNOS. When zeta potential falls (to less negative values) due to insufficient supplies of sulfates to maintain adequate negative charge around RBCs and other suspended particles, a compensatory mechanism sets in, for example through increased hepcidin synthesis and reduced erythropoietin-induced renewal of RBCs [83]. RBCs also experience a shorter lifespan when they are deficient in Ch-S [84], promoting a reduction in the hematocrit and therefore in blood viscosity.

It should be noted that the HS equation assumes homogeneity of the inner tube surface through which the fluid flows, but this situation might not apply at the capillary scale, where the electrical double layer (EDL) of vicinal water is strongly curved, polarized, anisotropic and surface conductive, and the interface is heterogeneous [85]. In addition, Bharti et al. [86] found that electroviscous effects on flow through cylindrical microchannels were stronger in shear-thinning liquids (for which viscosity decreases with increasing shear stress) than in Newtonian liquids (which display a linear response between applied shear stress and deformation rate). Human blood plasma is thought to behave as a shear-thinning liquid, just as do many biological solutions of protein and DNA [87].

Moreover, theoretical studies by Zhao et al. [78] indicate that the HS velocity of non-Newtonian fluids through cylindrical microchannels depends on the channel radius, an effect they attributed to nonlinear coupling among the electrostatics, channel geometry, and non-Newtonian hydrodynamics [78]. They also proposed a method for enhancement of electro-osmotic flows of power-law fluids under a combined DC and AC electric field [78], such as would be introduced by the beating heart.

Our final comment on the finding of EVSP-induced release of NO by endothelial cells relates to the biophysics of dissolved gases such as NO, CO, and H_2_S, which play complex signaling roles in blood homeostasis [68]. As noted in Section (The Exogenous Interfacial Water Stress (EIWS) theory of inflammation) above, Doshi and coworkers found that the size of a reduced-density water region near a hydrophobic surface depended strongly on the amounts and types of dissolved gases present [65], which could in turn influence the interaction between hydrophobic surfaces and the water in between them. They proposed further that lower-density water near a surface “should reduce their viscosity and boundary conditions for flow… from no-slip to partial or full-slip, which will have a significant impact on transport properties in small channels and on drag reduction in general” [65]. Their work maps directly to properties of the endothelial surface in capillaries, likely reflected as near frictionless flow or squeezing of RBCs through capillaries whose internal diameters of ca. 4–9 μm are nearly the same or even smaller than the average RBC diameter of ca. 7.8 μm [88].

### Biosulfates as enablers of healthy blood flow

With the remarks made in the previous two sections, we can begin to see how biological sulfates in the vascular system can influence important properties relating to interfacial water structure and blood flow. In this section we will continue our discussion with a more detailed consideration of how biosulfates, most notably sGAGs and Ch-S, may play essential roles in maintaining blood flow and electrokinietic properties. As discussed earlier [49], sulfurylated glycolipids, sGAGs, and sterol sulfates, including Ch-S, are ubiquitous in biological systems. These biosulfates generate the net surface charge density, as well as the specific surface charge density, and are determinants of bio-membrane viscosity and permeability [49]. They are also expected to have strongly electron-donating inductive effects on interfacial water.

**Fig. 4:**
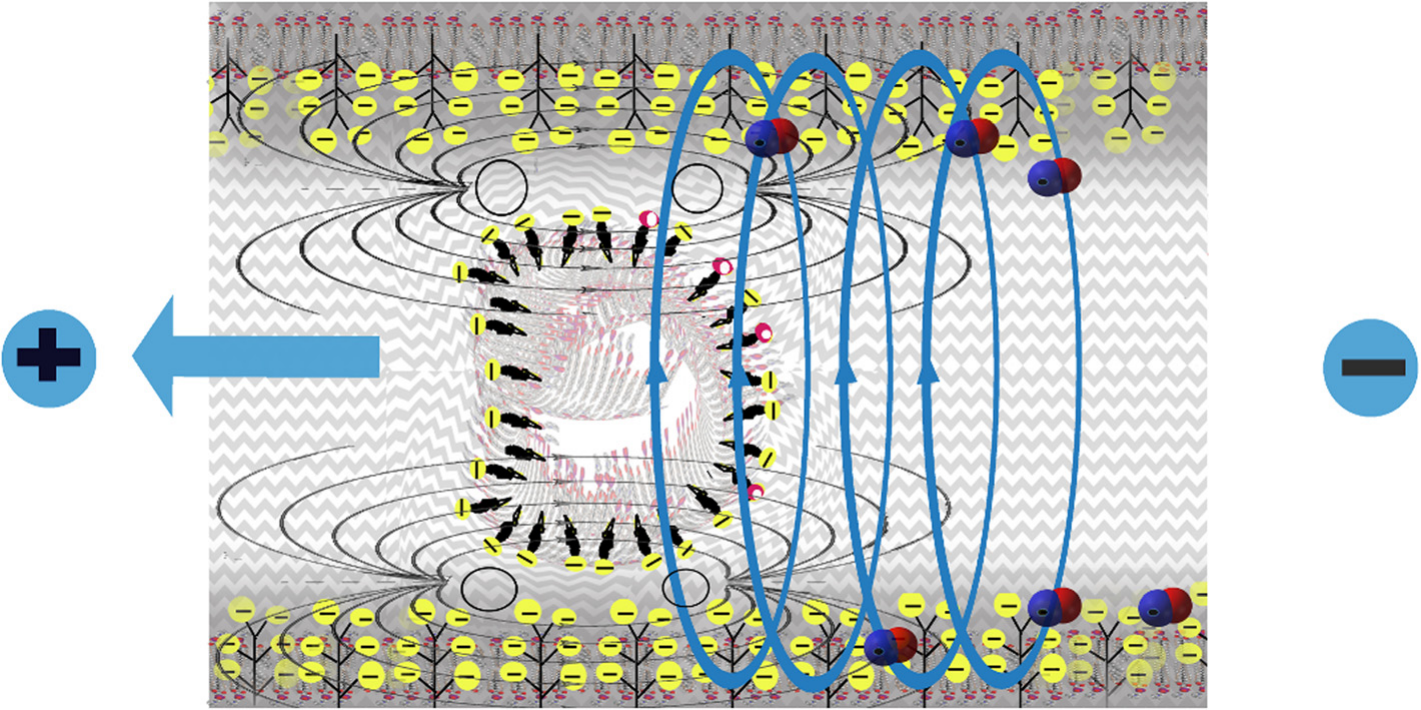
Schematic of a red blood cell moving through a capillary, propelled by the force field generated by the voltage drop between the artery and the vein. The moving RBC creates an electromagnetic field that induces the release of nitric oxide from the capillary wall. This relaxes the vessel and further promotes flow

To start our more detailed discussion, we propose that the HSPGs decorating the endothelial glycocalyx layer (EGL) of capillary endothelial cells provide a bottle-brush architecture similar to that reported by Banquy et al. for a synthetic mimic of the lubricating protein lubricin [89]. Figure 4 provides a schematic of the bottle-brush architecture of the glycocalyx. We suggest that the roughness of the bottle-brush, HSPG-decorated EGL induces capillary behavior of the blood, as implied by the molecular theory of capillarity [90]. This capillarity, when aided by (a) the amphiphilic Ch-S in endothelial and erythrocyte membranes and blood plasma, (b) a high (negative) ZP, and (c) high interfacial water tension, effectively facilitates the electrokinetic *pulling* of deformable RBCs through the microvasculature.

Sherman [91] has argued, through simple physical considerations based on Laplace’s equation of capillarity, that blood will flow out of capillaries into both the arteries and the veins when the surface tension is too high in the capillary, due to the inverse relationship between the size of the vessel and the pressure [91]. (We use the term “surface tension” here to refer to the amount of work needed to create an extra unit of liquid area at a liquid–vapor interface, while “interfacial tension” is understood to apply to the amount of work needed to create an extra unit of liquid area at a liquid-liquid or liquid–solid interface.) Interfacial water tension within a capillary lumen has both an endothelial water interface component and an erythrocyte water interface component, which may be represented by vectors normal to the endothelial and erythrocytic surfaces, respectively. We propose that, to avoid an intracapillary no flow or no reflow phenomenon, these vectors must dynamically negate each other’s force. We argue that anionic amphiphilic surfactants are essential to maintaining this balance. Cationic kosmotropes (water-structuring solutes) typically contract the surface (increase the surface tension), whereas anionic kosmotropes typically expand the surface (decrease the surface tension). The rate of surface tension lowering of anionic surface-active agents by electrolytes is largely determined by the charge of the added positive ion [92]. When an ionic surfactant is used to produce bubbles [93], the sign of microbubble charge is determined by the polar head of the surfactant.

More specifically, we propose that Ch-S acts as the key biosurfactant enabling healthy blood flow in the microvasculature, fulfilling a role similar to that played by sodium dodecyl sulfate (SDS) in SDS- polyacrylamide gel microelectrophoresis. In recent high-speed video studies of aqueous droplets moving electrophoretically through oil, Hamlin et al. found that, at relatively low or high surfactant concentration, the droplets responded to reversal of the electric field by quickly accelerating and then decelerating in the opposite direction, “concurrent with a transient rearrangement of tracer particles on the droplet’s surface” [94]. Of note, one of the surfactants, SDS, is structurally similar to the amphiphilic sterol sulfate, Ch-S. Hence, the role we have postulated for Ch-S in enhancing microvascular blood flow might involve facilitation of a metastable Marangoni-type fluctuation in mass transfer along the interface due to changing surface tension gradients. This effect would enable erythrocytes to flow, in conjunction with ELF EM energy provided under our hypothesis, to the EGL target by the EVSP. The thorough studies of Colacicco dealing with the role of SDS in the electrical potential at an oil–water interface [95] also have implications with respect to how Ch-S might be involved in generation and maintenance of the cellular resting electrical potential, action potential, electrokinetics, and zeta potentials.

Before closing this section, it is worth noting that Davidson and Seneff [49, 50] have proposed yet another way in which sulfur insufficiency may give rise to impaired blood flow. While the cardiovascular complications of diabetes are often attributed to loss of nitric-oxide-mediated vasodilation, an alternative pathophysiology was suggested, involving impaired microvascular perfusion due to (a) decreased fluidity and deformability of the RBC membrane related to biosulfate deficiency in the endothelial glycocalyx layer (EGL); and (b) increased capillary endothelial interfacial (surface) tension [49, 91]. This proposed mechanism disagrees with Burton’s basic assumption of flow being arrested by active arteriolar constriction [91–96].

If adequate supplies of sulfate are essential for maintaining blood vessel health, then the organism needs the capability to redistribute existing sulfate resources wherever needed in the cardiovascular system and to convert (oxidize) more-reduced sulfur compounds to sulfate if other sources of sulfate are insufficient. As mentioned above, we previously proposed an eNOS- and sunlight-catalyzed mechanism as a normal route for sulfate biosynthesis [16]. In the following three sections of the present paper, we will consider sulfate redistribution mechanisms and alternative, emergency sulfate synthesis pathways that generate, as side effects, various forms of blood vessel damage and other symptoms associated with cardiovascular disease.

### Extracellular matrix sGAG turnover as a mechanism for sulfate redistribution

In the preceding section, we presented our hypothesis that Ch-S plays an essential role in enabling healthy blood flow through the capillaries. In this section, we discuss the deposition of cholesterol in the cardiovascular system when insufficient sulfate is available to make Ch-S. We will then propose a mechanism by which capillary levels of Ch-S can be replenished, in event of a deficiency, by recruitment of sulfate released by sGAG turnover in the extracellular matrix (ECM) of atherosclerotic regions.

Cholesterol is made by all cells in the body, but, being lipophilic, does not easily migrate from intracellular synthesis or storage sites to the plasma membrane. Sulfurylation will make the molecule amphiphilic, enabling it to facilitate blood flow through capillaries as discussed above, and reducing or eliminating the need to package cholesterol inside a lipoprotein particle for transport within the water-based cell cytoplasm. In addition, Ch-S shows a 10-fold higher rate of exchange between two lipid membranes than does cholesterol [97], which can be expected to greatly accelerate uptake by HDL, the lipoprotein that can remove cholesterol from the atheroma.

ApoE is the lipoprotein that escorts Ch-S from the Golgi apparatus to the plasma membrane, and ultimately delivers it to HDL-A1 [98]. ApoE’s antioxidant properties probably allow it to protect Ch-S in transit from oxidative damage. Indeed, ApoE promotes both cholesterol egress from the macrophages in the plaque and sulfurylation of the glycocalyx [98], and this hints that Ch-S might play an intermediary role. SULT2B1b is the specific sulfotransferase that sulfurylates cholesterol. It also sulfurylates oxysterols such as 7α-hydroxycholesterol and 27-hydroxycholesterol with nearly the same affinity [99]. Sulfurylation significantly reduces the cytotoxicity of these oxysterols, which are present in the atheroma. It is plausible that oxysterols, which are more water soluble than cholesterol itself, are exploited to deliver sulfate to the extracellular matrix.

Platelets also tend to accumulate in cardiovascular plaque, and, if supplied with activated sulfate in the form of PAPS, they exuberantly produce Ch-S [100]. They release it, specifically, to HDL-A1 [98]. They will accept no other lipid particle as the recipient. Significantly, HDL-A1 is the only form of HDL that is atheroprotective [101].

There is strong evidence from studies on plaque formation that cholesterol is actively recruited into the atheroma. Contrary to the widely perceived model that cholesterol passively enters the artery wall from the luminal side, a provocative paper published by Subbotin in 2012 [102] proposed that cardiovascular disease is initiated by neovascularization of the tunica intima, and that cholesterol is actively recruited directly from the newly formed capillaries. He also argues that this process begins early in life. We propose that this cholesterol is accumulated as a reserve supply, which will be conjugated with sulfate to form Ch-S as soon as sulfate becomes available.

A study on the export of cholesterol from macrophages in cardiovascular plaque into plasma discovered that only 40 % of the cholesterol was extracted into HDL-A1, and a significant percentage of the rest, surprisingly, was extracted by serum albumin [103], despite the fact that the cholesterol acceptor activity for albumin is 280-fold lower than that of apoA1. This supports our hypothesis that it is sulfoconjugated prior to export. Ch-S and DHEA sulfate bind tightly to albumin, in direct contrast to their unsulfurylated counterparts [30, 104]. Furthermore, increased uptake by albumin was associated with increased risk to cardiovascular events [103]. We hypothesize that this may reflect Ch-S depletion in albumin. DHEA sulfate, but not DHEA, inhibits NF-κB synthesis, suggesting that sulfate deficiency is a driver of inflammation [105].

Clearly, insufficient sulfate supplies will impair production and transport of Ch-S to the microvasculature to fulfill its proposed surfactant and electrophoretic functions as described in Section (Biosulfates as enablers of healthy blood flow) above. In the remainder of this section, we will discuss our hypothesis for a mechanism by which sulfate needed for Ch-S can be harvested by taking advantage of sGAG turnover in the extracellular matrix (ECM) of the cardiovascular system.

The sGAG-rich glycocalyx plays an important regulatory role in vascular permeability [106]. The sulfurylation patterns in these complex sugar chains are intricate and tightly regulated [107]. Changes in vascular glycocalyx structure regulate cellular adhesion, proliferation, migration and signalling in association with cardiovascular disease [108]. Extracellular proteoglycans, including sGAGs and the sulfate-free hyaluronan (HA), maintain hydration, mechanical properties, and concentration gradients of critical signalling molecules [109].

**Fig. 5:**
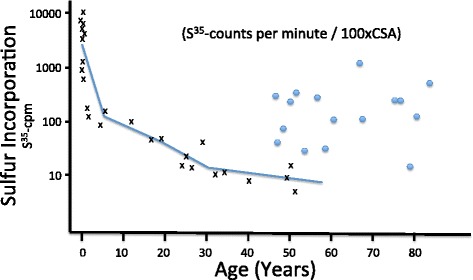
Sulfur Incorporation into GAGs with Age. Incorporation of ^35^Sulfur into chondroitin sulphuric acids (CSA) of the connective tissue of normal human aortae (x) and atherosclerotic human aortae (o) in *in vitro* experiment. (Redrawn from [110], with permission from Elsevier)

A remarkable series of investigations by Hauss et al. in the 1960’s [110, 111] measured the uptake of sulfur into sGAGs in the artery wall and its biological half-life as a function of age. Their studies revealed that the amount of sulfur that is taken up by the sGAGs is highest very early in life and decays exponentially over time, and that the retention cycle also decreases with age. However, the incorporation of sulfur into sGAGs in diseased regions where cardiovascular plaque accumulates is several *fold* higher than what would be predicted based on the age curve (see Fig. 5). The simplest explanation is that the atheroma is actively engaged in accumulating sGAGs for redistribution.

Hyaluronan (HA) concentration is increased dramatically in association with smooth muscle cell migration and proliferation following arterial injury, along with that of versican, a chondroitin-sulfate-enriched proteoglycan [112]. Both HA and versican are found in high concentrations in atherosclerotic plaque, particularly in association with the trailing edge of migrating smooth muscle cells. A postmortem study of stented arterial regions showed that chondroitin sulfate and HA were the predominant components of the neointimal proteoglycans [113], and vessel injury at the stent site was proposed as a factor inducing this buildup. Like the sulfate and sulfamidate groups of sGAGs, the multiple carboxylate groups attached to HA could also effect a structuring of the surrounding water [50], resulting in a clear solute free zone surrounding the smooth muscle cells [112]. Versican is tethered to the HA matrix, and when HA is degraded, the versican is also released into the surrounding space.

A pericellular matrix gel forms around these aggregated HA-versican fibrillar complexes produced by migrating smooth muscle cells but then appears to dissolve into the medium about one hour later [112]. The main purpose of the matrix may well be to produce materials (HA and versican) that can be degraded to enable redistribution of the building blocks to distant cells. This degradation can be accomplished with NO and/or one of its reactive oxidant derivatives. NO gas degrades heparin and heparan sulfate (HS) at concentrations as low as 400 ppm. NO oxidizes rapidly to HNO_2_, which can also break down HS chains that are attached to membrane proteins such as syndicans and glypicans [114]. On the other hand, peroxynitrite (ONOO−), the product of reaction of NO with superoxide, is particularly effective at breaking down HA, which will permit the release and redistribution of fragments of both HA and the attached chondroitin sulfate chains, thereby providing a sulfate source to replenish depleted supplies in the myocardial microvasculature.

In support of this hypothesis, Kruse et al. have reported that significant decreases in HS content and increases in the amounts of chondroitin sulfate/dermatan sulfate relative to HS are observed in association with elevated cholesterol levels in human aorta [115]. Thus, there is an inverse relationship between cholesterol retention and heparan sulfate supply. Furthermore, the observed redistribution of sGAG content away from HS (which inhibits smooth muscle cell proliferation) toward chondroitin sulfate-rich GAG is consistent with a move toward enabling smooth muscle proliferation to free up stored sulfate for possible use in generating Ch-S.

An in-depth discussion of the connection between diabetes and CVD is beyond the scope of this paper, but it is worth noting that the advanced glycation end products (AGEs) associated with hyperglycemia and diabetes also trigger processes that promote inflammation and smooth muscle cell proliferation, leading to generation of reactive oxygen species (ROS) and induction of an atherosclerotic profile [116]. Oxidative stress is a well-established risk factor for vascular disease [117]. H_2_S, in addition to its antioxidant effects, can protect from diabetes by increasing the cellular levels of phosphatidylinositol 3,4,5-trisphosphate (PIP3), a positive regulator of glucose metabolism [118].

### Glutathione and GGT

In the preceding section, we discussed a possible mechanism by which the cardiovascular system could relieve a shortage of Ch-S and avoid a crisis in capillary blood flow by redistributing sulfate from sGAGs. We will now turn our attention to ways in which the total biosulfate supply can be replenished by “emergency” conversion of less-oxidized sulfur compounds. As noted in Section (Evidence for a role of sulfur deficiency in atherosclerosis) above, we have proposed elsewhere [16] that, under normal, non-inflammatory conditions, sulfate synthesis from thiol- and sulfane-type substrates (HS^−^, glutathione persulfate, thiosulfate, etc.) in RBCs, platelets, and other endothelial cells can be facilitated by eNOS and sunlight. In the present section, we will consider other sulfate biosynthesis routes from thiols that can be utilized if sunlight exposure is inadequate or if eNOS function is impaired through exposure to environmental toxicants.

Sulfur homeostasis in plants is a good model for sulfur management in biological systems more generally [119]. Plants utilize glutathione (GSH) as the main form of reduced sulfur storage [120]. The concentration of GSH is much higher than the concentration of free cysteine in plant cells, and this may be because it is less reactive than free cysteine, and therefore more attractive for sulfur storage. The same may be true for animals. A study exposing rats to a GSH synthase inhibitor resulted in a three-fold reduction in tissue GSH along with indicators of oxidative stress and hypertension. This was linked to reduced synthesis of nitric oxide (NO) in the vascular wall [121].

This relates to the observation, now thoroughly confirmed, that elevated γ-glutamyl transferase (GGT), an enzyme that breaks GSH down into its component amino acids, is an outstanding serum marker for a host of modern diseases, including liver dysfunction, cardiovascular disease [122, 123], digestive disorders, Alzheimer’s disease, metabolic syndrome, obesity, diabetes, musculoskeletal disorders, cancer and death [124–127], with high significance. Ischemia depletes glutathione in both liver [128] and brain [129]. Elevated serum GGT strongly predicts carotid plaque, which, in turn, is highly predictive of future cardiovascular disease [130].

While GSH is mostly known as an antioxidant, extracellular GSH can exhibit a pro-oxidant effect in the presence of GGT [131]. GGT, by splitting off glutamate, produces cysteinyl glycine, which is more acidic than glutathione (pKa 6.4 vs. 8.56, respectively) [132], making it more reactive in converting Fe^+3^ to Fe^+2^. However, another factor that may be at play is the fact that the carboxylate group of the glutamate residue in GSH chelates with iron, reducing its bioavailability for reaction with the sulfhydryl group of the cysteine moiety [133]. GGT separates the glutamate from the other two amino acids, facilitating iron reduction to Fe^+2^ and subsequent redox reactions. In the presence of free iron (Fe^+3^), this can lead to the Fenton reaction and subsequent production of superoxide.

While the research literature does not provide any explanation for a possible beneficial effect of this superoxide production, we propose that it is necessary in order to promote oxidation of the sulfur atom in cysteinyl-glycine to sulfate. We hypothesize that cysteine is liberated from GSH for subsequent breakdown to supply sulfur as a substrate for sulfate production. In oxidative cysteine catabolism, cysteine dioxygenase oxidizes the sulfur in cysteine, leading ultimately to the production of taurine, pyruvate and sulfate [134]. Cysteine might also be converted to homocysteine and subsequently homoycysteine thiolactone, which can also be substrate for sulfate synthesis through a pathway requiring vitamin C and vitamin A as well as superoxide [17]. Elevated serum homocysteine is a strong risk factor for cardiovascular disease [14, 135–137]. In fact, homocysteine is known to induce vascular oxidation damage [138, 139]. We propose that the need for homocysteine and for superoxide is driven by the insufficient supply of Ch-S to the tissues.

Another available pathway is to produce hydrogen sulfide gas (H_2_S) from cysteine by enzymatic action of cystathionine γ lyase (CSE). H_2_S has only relatively recently been recognized as a signaling gas on par with nitric oxide [68], and it has been shown to induce vascular relaxation as well [140]. H_2_S offers similar protective effects as NO without the issue of reacting with superoxide to produce the toxic metabolite ONOO^−^ [141–143]. H_2_S in small concentrations promotes angiogenesis and protects against both atherosclerosis and hypertension [144]. CSE is present in both vascular smooth muscle cells (SMCs) [145] and blood lymphocytes [68]. It has only recently been realized that H_2_S can be used by mammalian mitochondria as an energy source during times of hypoxia.

In experiments on vascular smooth muscle cells, it was shown that stress-induced calcium entry causes CSE to migrate to the mitochondria, where cysteine is already available in high concentration [146]. These authors wrote: “The notion that eukaryotes do not need H_2_S in the energy metabolism process should be revised. In SMCs, mitochondrial CSE translocation and H_2_S production confer resistance to hypoxia by increasing ATP synthesis.” We argue that, in addition to this, it is also an important substrate for sulfate synthesis. The CSE enzymatically produces H_2_S from cysteine, along with pyruvate as a by-product, another energy source that also reduces electron leakage. H_2_S is quickly oxidized by the electron transport chain to sulfate and thiosulfate [146], and helps maintain ATP levels in a hypoxic environment. Thiosulfate can be converted to sulfate in a reaction catalyzed by thioredoxin reductase, which can be used in certain microorganisms and possibly even in mammalian cells as an energy source for further ATP production, restoring H_2_S and completing the cycle [147].

We can now explain the refractory statin resistance observed in the context of certain highly inflammatory conditions such as lupus or kidney failure. Here, cardiovascular mortality is increased by up to 33 to 50 fold [148], yet, despite this, serum LDL is often not elevated, or may even be abnormally low. A recent investigation revealed that, in these cases, smooth muscle cells in the aorta use coenzyme A reductase to synthesize their own cholesterol for local storage, and these cells show resistance to statin therapy compared to hepatic cells [3]. This suggests to us that the cholesterol is desperately needed to maintain blood stability.

Although supportive evidence of our hypothesis is strong, further *in vitro* studies specifically addressing the concept of cholesterol sulfate’s role in protection from atherosclerosis are needed. In addition, clinical trials involving dietary modification to include enrichment in sulfur-containing amino acids and other sulfur sources, along with increased sun exposure to the skin and avoidance of environmental toxicants, will be an important step towards validating our hypothesis.

## Conclusion

In this paper, we have put forward the novel hypothesis that impaired cholesterol sulfate supply to the heart is the key pathology behind atherosclerosis. This explains why regions of the artery wall with increased sulfate uptake and recycling in the endothelial glycocalyx also have excess cholesterol storage. We offer key insight into the importance of sulfates to vascular health through arguments based on the unique properties of water and the EIWS theory of disease. We hypothesize that CYP enzyme disruption by multiple environmental toxicants may be a significant factor in atherosclerosis, as eNOS contains a CYP structural motif that is likely essential for sulfate synthesis. We argue that the accumulation of cholesterol in the artery wall is a reflection of impaired cholesterol transport due to low bioavailability of sulfate to the vasculature. The constant remodeling of the extracellular matrix in occluded arterial regions reflects the need to break down and distribute to the capillaries the sGAGs that must populate the glycocalyx to maintain vascular health and enable blood flow. Supportive evidence comes from the observation that the differentiation, mobilization, and proliferation of SMCs in the artery wall is accompanied by rapid synthesis and release of chondroitin sulfate.

While the view that atherosclerosis is an inflammatory disease is gaining in popularity today [149], anti-inflammatory pharmaceutical drugs increase the risk to heart disease [150]. Elevated homocysteine is a strong risk factor for atherosclerosis, yet attempts to correct it have met with failure. Administration of B vitamin supplements in an attempt to reduce serum homocysteine has been effective, but did not result in any clinical improvement in cardiac disease risk [151–153]. All of these seemingly contradictory results can be explained if we assume the point of view that the inflammation is needed to induce superoxide production, which will allow sulfate production with homocysteine, H_2_S, and superoxide as substrates. Any attempt to reduce either the superoxide or the homocysteine levels will further impair the supply of sulfate to the capillary glycocalyx.

Platelet complement activation, a key factor in atherogenesis, leads to inflammation and thrombosis, both of which may be necessary for the intense regeneration of sulfate at the injury site and its mobilization, in order to restore vascular health. A recent review article on platelet activation summarized as follows: “We hypothesize that platelets focus complement activation to sites of vascular injury, and that interactions between platelets and the complement system contribute to acute and chronic inflammation and thrombosis.” ([154], p. 5). We have argued here that the inflammation renews sulfate supplies and the thrombosis reduces viscosity. Vascular flow is improved, and this leads to nitric oxide production, mediated by EVSP. Nitric oxide reaction products break down and redistribute locally-produced sGAGs.

Based on a review of the literature, this paper describes several novel means by which the biosulfates play essential roles in enabling microvascular blood flow, including their impact on the hemorheological parameters - the zeta potential, streaming potential, capillarity, viscosity, and interfacial tension, without which a “no flow” situation would surely exist. It argues that lifestyle is causal and simple lifestyle changes may have profound benefits. These include eating strictly organic sulfur-rich foods and making special efforts to avoid environmental toxicants and to promote sun exposure to the skin without sunscreen.

## Abbreviations

25HCS: 25-hydroxycholesterol sulfate
AGEs: Advanced glycation endproducts
CDs: Coherence domains
CSE: Cystathionine gamma lyase
CYP: Cytochrome P450
Ch-S: Cholesterol sulfate
ECM: Extracellular matrix
EDL: Electrical double layer
EDRF: Endothelial-derived relaxing factor
EGL: Endothelial glycocalyx layer
EIWS: Exogenous interfacial water stress
EVSP: Electrokinetic vascular streaming potential
EZ: Exclusion zone
GAGs: Glycosaminoglycans
GGT: Gamma glutamyl transferase
GSH: Glutathione
HA: Hyaluronan
HDL: High density lipoprotein
HS: Heparan sulfate
HSPGs: Heparan sulfate proteoglycans
LDL: Low density lipoprotein
MI: Myocardial infarction
NDSTs: N-deacetylase/N-sulfotransferases
NF: Necrosis factor
NO: Nitric oxide
PAPS: 3′-phosphoadenosine-5′-phosphosulfate
PDGF: Platelet derived growth factor
PPAR: Peroxisome proliferator-activated receptor
RBCs: Red blood cells
ROS: Reactive oxygen species
SDS: Sodium dodecyl sulfate
SMCs: Smooth muscle cells
VEGF: Vascular endothelial growth factor
eNOS: Endothelial nitric oxide synthase

## Footnotes

1 Throughout this paper, as a significant nomenclatural point, we have adopted use of the words “sulfurylate” and “sulfurylation,” in strong preference to the words “sulfate”, “sulfation”, and “sulfonation”. Biochemically, sulfate ester and sulfamidate formation occur most commonly via sulfuryl group transfer. Use of the term “sulfation” is likely to only apply to *in vitro* electrophilic aromatic substitution and olefinic addition reactions, which involve carbocation intermediates. Whereas *in vivo* only 3 oxygen atoms transfer with the *sulfuryl* group to an acceptor ROH or RNH2 molecule to produce a sulfate ester or sulfamidate, respectively. Such a mechanism of “*sulfuryl*” group transfer via “sulfurylation” has important mechanistic implications, which are highly-analogous to “phosphoryl” group transfer via “phosphorylation” in organophosphorus chemistry.
